# Detecting the First Hydration Shell Structure around Biomolecules at Interfaces

**DOI:** 10.1021/acscentsci.2c00702

**Published:** 2022-09-06

**Authors:** Daniel Konstantinovsky, Ethan A. Perets, Ty Santiago, Luis Velarde, Sharon Hammes-Schiffer, Elsa C. Y. Yan

**Affiliations:** † Department of Chemistry, Yale University, New Haven, Connecticut 06520, United States; ‡ Department of Molecular Biophysics and Biochemistry, Yale University, New Haven, Connecticut 06520, United States; § Department of Chemistry, University at Buffalo, Buffalo, New York 14260, United States

## Abstract

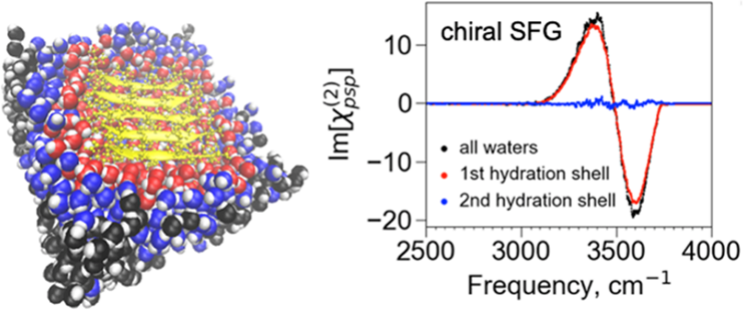

Understanding the role of water in biological processes remains a central challenge in the life sciences. Water structures in hydration shells of biomolecules are difficult to study *in situ* due to overwhelming background from aqueous environments. Biological interfaces introduce additional complexity because biomolecular hydration differs at interfaces compared to bulk solution. Here, we perform experimental and computational studies of chiral sum frequency generation (chiral SFG) spectroscopy to probe chirality transfer from a protein to the surrounding water molecules. This work reveals that chiral SFG probes the first hydration shell around the protein almost exclusively. We explain the selectivity to the first hydration shell in terms of the asymmetry induced by the protein structure and specific protein–water hydrogen-bonding interactions. This work establishes chiral SFG as a powerful technique for studying hydration shell structures around biomolecules at interfaces, presenting new possibilities to address grand research challenges in biology, including the molecular origins of life.

## Abstract 2

Experiments and computation show that vibrational chiral sum frequency generation spectroscopy is sensitive to the vibrational structure of water in the first hydration shell around biomolecules.

## Introduction

Water, interfaces, and homochirality are chemical hallmarks of biological life. However, most water molecules in the hydration shells of biomolecules are missing from structural data acquired using standard techniques such as X-ray crystallography and electron microscopy. Hydration shell water molecules are very difficult to study due to their rapid dynamics and exchange with the bulk solvent. Nevertheless, the motion and arrangement of these water molecules can significantly modulate the structures and functions of biomolecules.^1−5^ Moreover, the chemical behavior of water and biomolecules also changes near interfaces.^6^ Aqueous interfaces are ubiquitous in biological systems and are chemically distinct from bulk aqueous environments because of the presence of chemical asymmetries, notably the termination of bulk water hydrogen bonding (H-bonding) networks. Hydration shell structures around biomolecules will therefore change dramatically as the biomolecule approaches an interface. Although NMR,^7−9^ neutron scattering,^4,10−15^ terahertz spectroscopy,^16,17^ vibrational circular dichroism,^18^ and Raman optical activity^19^ are useful in probing aspects of the structure and dynamics of hydration of biomolecules, none of these methods are interface-specific. Thus, studying local water interactions in hydration shells of biomolecules *in situ* and under ambient conditions at interfaces remains a significant experimental challenge.

Here we show that chiral sum frequency generation (chiral SFG) spectroscopy can tackle this challenge by offering remarkable selectivity to probe water structures in the first hydration shell of a biomolecule. We demonstrate that the method can suppress signals of water molecules outside the first hydration shell at interfaces and in the bulk solution. Chiral SFG reveals the O–H stretching vibrational frequencies of water, thus enabling studies of local H-bonding interactions between water and biomolecules. This extraordinary capability to probe local H-bonding structures of water in the first hydration shell of biomolecules *in situ* at interfaces will support the pursuit of a central challenge in the life sciences: elucidating the role of water in biomolecular function.

Chiral SFG is an interface-specific, chiral-selective vibrational spectroscopy. For over a decade, chiral SFG has been developed as a tool for the characterization of biomolecules in chiral secondary and higher-order structures at interfaces, such as on lipid membranes and polymer surfaces.^20−28^ In 2017, Petersen and co-workers first detected chiral SFG signals from a chiral assembly of water molecules hydrating DNA.^29^ Our group later showed that chiral assemblies of water molecules also form around proteins and that the chirality of the water assemblies correlates with the chirality of the protein.^23,30−32^ These studies show the promise of using chiral SFG to probe water structures in hydration shells of biomolecules at interfaces. Nonetheless, one outstanding question remains. What are the structural and dynamical properties of the chiral supramolecular assemblies of achiral water molecules that generate a chiral SFG response? This question needs to be answered before chiral SFG can be fully utilized to study the hydration of biomolecules at the fundamental level.

**Figure 1:**
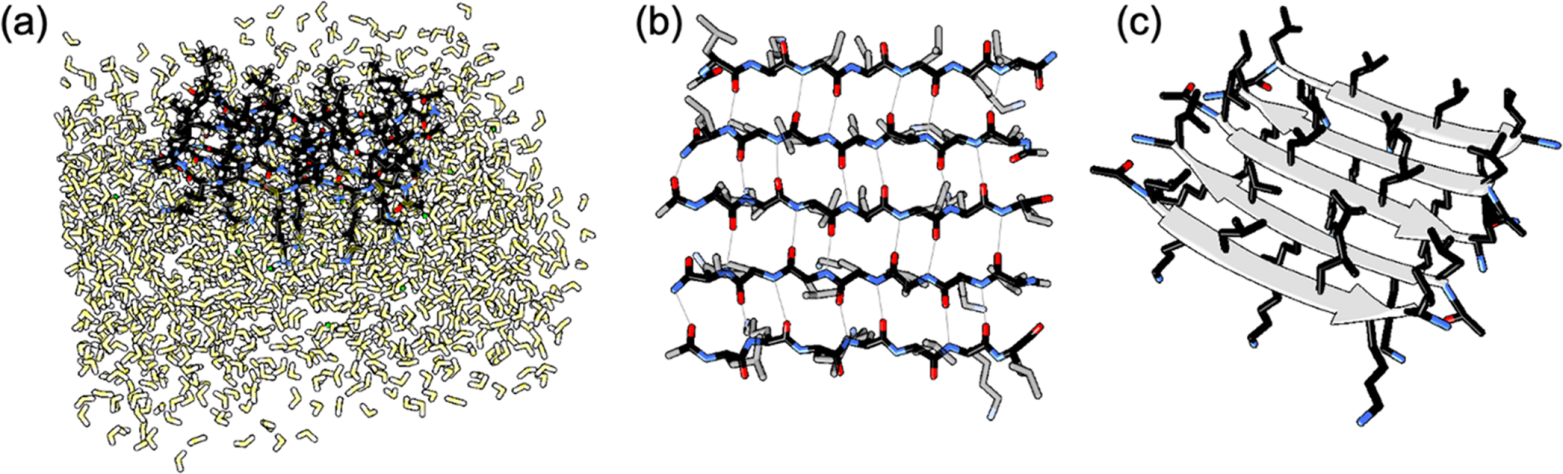
Molecular system used for the computational analysis of the chiral SFG response of water around a protein. (a) The formation of an aniparallel β-sheet from an LK_7_β pentamer (black) at the vacuum–water interface. The protein is surrounded by ∼1340 water molecules (yellow). (b) A top view of the LK_7_β pentamer, with the backbone highlighted in colors and the side chains represented in gray. The aqueous phase is below this structure. (c) A side view of the same pentamer, with side chains highlighted in colors and the backbone represented in gray. The polar lysine residues are directed into the water, and the nonpolar leucine residues are directed into the vacuum. In panels b and c, hydrogen atoms have been removed for clarity.

We seek to answer this outstanding question using a model system, namely, LK_7_β (Ac-LKLKLKL-NH_2_), which is an amphiphilic peptide that assembles into antiparallel β-sheets at interfaces (Figure 1).^23,30^ We perform experiments to measure the chiral SFG response from water hydrating LK_7_β. We then build atomistic models of the antiparallel β-sheet at an aqueous interface and simulate the chiral SFG response of water with molecular dynamics (MD). Based on these simulations, we dissect the overall chiral SFG signals into those generated by subsets of water molecules in the system. Such computational investigations reveal correlations between local H-bonding interactions, the O–H stretching frequency, and the intensity of the chiral SFG response. More importantly, chiral SFG is demonstrated to possess exclusive selectivity for water structures in the first hydration shell. We further analyze how the mobility of water molecules is related to their effectiveness in producing chiral SFG signals and how the orientations of water dipoles arrange around the protein at the interface. These analyses uncover the molecular origin of the remarkable selectivity of the chiral SFG method to the first hydration shell based on established symmetry-based chiral SFG theory.^33,34^ We discuss the implications of these findings in the context of recent progress in the study of biomolecular hydration using various methods in the field. We conclude that chiral SFG offers the unique capability to probe water vibrations in hydration structures of biomolecules at interfaces with selectivity to the first hydration shell.

## Results and Discussion

### Comparison of Experimental and Computational Spectra

**Figure 2:**
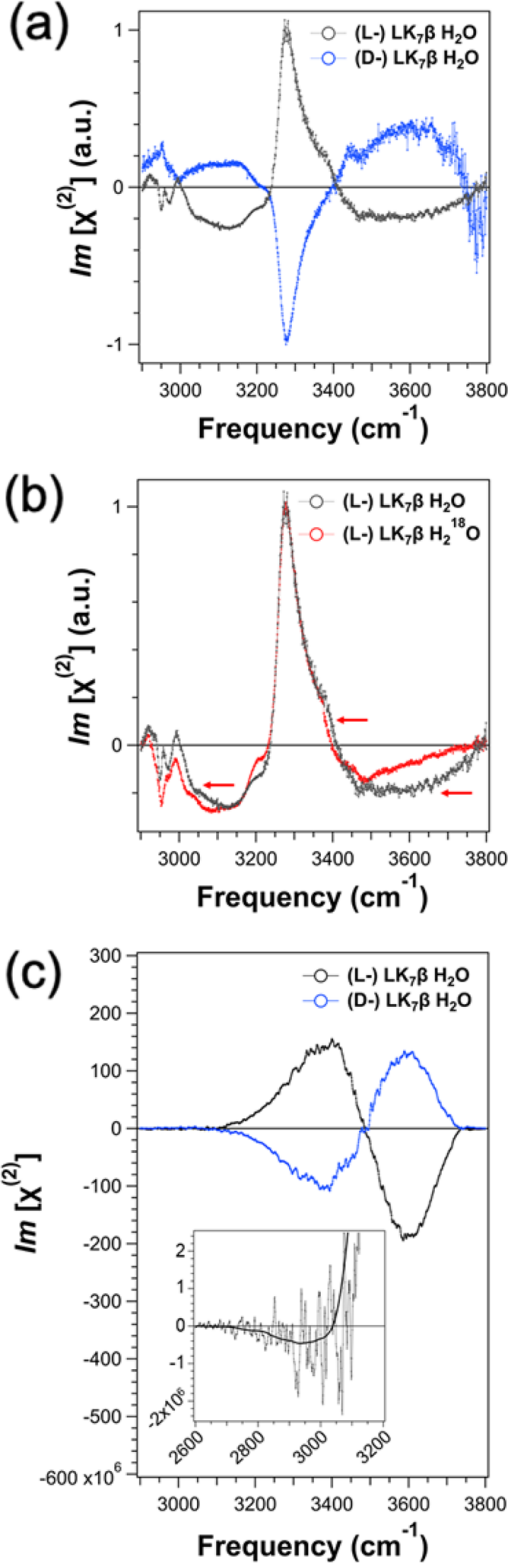
Experimental and computational chiral SFG spectra containing spectral contributions due to water. (a) Full experimental spectrum in the N–H/O–H region of (L-) LK_7_β and (D-) LK_7_β. (b) Experimental H_2_O and H_2_^18^O spectra of the N–H/O–H region of a hydrated film of LK_7_β, which reveals peaks due to water (red arrows). (c) Calculated spectra of the O–H stretch response for (L-) LK_7_β and (D-) LK_7_β. The inset shows a small low-frequency peak in the (L-) LK_7_β spectrum (raw data and smoothed curve). The low-frequency peak is statistically significant compared to the spectral baseline noise (*p* = 0.037, two-sided *t*-test).

We start our investigations by obtaining experimental chiral SFG spectra of water around the antiparallel β-sheet LK_7_β (Figure 1) and comparing the experimental spectra with the computational spectra simulated with MD (Figure 2). Our experimental setup consists of a hydrated film of (L-) or (D-) LK_7_β deposited on a quartz surface for heterodyne detection, as previously described.^31^ Compared with our previous studies,^31^ the experimental chiral SFG spectrum extends the spectral region beyond 3400 cm^–1^ (Figures 2a and 2b) for direct comparison with the simulated spectra (Figure 2c). Figure 2a shows that the phase of the chiral SFG signal flips when (L-) LK_7_β is replaced with (D-) LK_7_β. The experimental spectra consist of N–H and C–H stretching peaks from the protein as well as O–H stretching peaks from water. The C–H stretching modes in LK_7_β only appear below 3000 cm^–1^. Because the N–H and O–H stretching peaks overlap at frequencies above 3000 cm^–1^, we identified the water O–H stretching contributions using H_2_^18^O labeling (Figure 2b). Isotopic substitution with H_2_^18^O redshifts the O–H stretching frequencies by roughly 12 cm^–1^ (Figure S2 and Table S1).^35^ This identifies peaks at 3151, 3366, and 3555 cm^–1^ as coming from the O–H stretch (see Table S1). Other spectral features (>3000 cm^–1^) do not redshift upon H_2_^18^O substitution and therefore are assigned to the N–H stretching modes of LK_7_β (Figure S2 and Table S1). H_2_^18^O labeling reveals broad spectral features of water that span three spectral regions: 3050–3150, 3300–3400, and 3500–3800 cm^–1^. The features that appeared around 3050–3150 and 3300–3400 cm^–1^ were previously observed and assigned to the O–H stretch of water solvating LK_7_β.^30,31^ This experiment therefore identifies additional contributions of water in the high-frequency region of 3500–3800 cm^–1^.

We calculated the chiral SFG spectrum of the water around (L-) LK_7_β (Figure 2c) using MD simulations. The model system consists of five antiparallel strands of the LK_7_β protein surrounded by ∼1340 water molecules and placed at the vacuum–water interface, where the hydrophilic lysine side chains point toward the aqueous phase and the hydrophobic leucine side chains point toward the vacuum (Figure 1). Our calculations of the vibrational spectra rely on Skinner’s mapping approach, which is based on density functional theory (DFT) calculations linking local electric fields to O–H vibrational frequencies and transition dipoles.^36−40^ The computational spectra (Figure 2c) therefore exhibit water O–H peaks but not protein N–H peaks. To calculate the chiral SFG signal, we calculated an orthogonal (“chiral”) second-order susceptibility tensor component, which is nonzero only for a chiral interface. The more commonly detected and simulated SFG response from achiral interfaces originates from nonorthogonal components, such as those calculated in the Skinner group’s work^36,38−40^ (see Methods for technical details). As shown in our previous study,^31^ the phase of the computational chiral SFG spectrum flips when the chirality of the protein is switched from the (L-) form to the (D-) form (Figure 2c), agreeing with the experimental observation (Figure 2a). Each spectrum contains two major features: a positive peak at around 3400 cm^–1^ and a negative peak at around 3600 cm^–1^ for (L-) LK_7_β; for (D-) LK_7_β, the same pair of peaks have the opposite phase. These two peaks appear to agree with the experimentally observed water contribution in the two high-frequency regions of 3300–3400 and 3500–3800 cm^–1^ (Figure 2a).

The computational spectrum seemingly does not show any low-frequency spectral features at 3050–3150 cm^–1^ that match the experimental observation. Nonetheless, careful examination of the computational spectrum (inset, Figure 2c) reveals a small feature at around 3000 cm^–1^ just above the noise level but statistically significant (*p* = 0.037 in a two-sided *t*-test when the signal between 2850 and 3050 cm^–1^ is compared to the baseline between 3800 and 4000 cm^–1^). Further analysis of the chiral SFG response shows that O–H stretches at this low frequency can originate from water forming strong H-bonds with LK_7_β, which will be discussed in detail below. It is important to note that our calculations did not take Fermi resonances into account, and Fermi resonance with the first overtone of the water bending mode may contribute to the low-frequency peak.^41^ Overall, the computational spectra (Figure 2c) capture the most important features of the experimental spectra (Figure 2a), showing the chiral SFG response of the O–H stretching modes of water molecules hydrating LK_7_β.

### Chiral SFG Signal of Water is Primarily Generated from the First Hydration Shell

**Figure 3:**
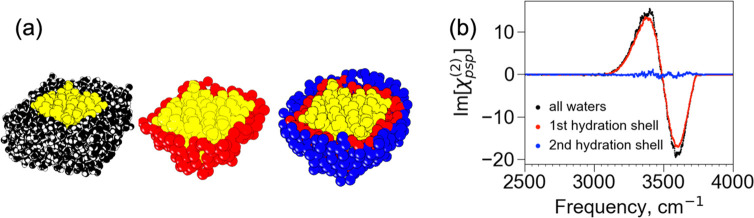
Almost all the chiral SFG response of water originates from the first hydration shell around the protein. (a) The model systems used for this analysis contain the LK_7_β pentamer protein (yellow) together with different subsets of water molecules: all water (black), the first hydration shell (red), and the second hydration shell (blue). (b) Calculated spectra of the O–H stretch response from all water molecules (black), the first hydration shell (red), and the second hydration shell (blue).

Our current study centers on dissecting the overall chiral SFG responses of water molecules into those that interact with various parts of LK_7_β, *e.g*., the first and second hydration shells. The key to the analysis is the use of the Voronoi tessellation algorithm (see Methods). This algorithm divides the entire simulation system into nonoverlapping three-dimensional compartments, where each compartment is exclusively occupied by one atom. The boundary of each compartment is delineated using Voronoi tessellation. These boundaries can then be used to identify all immediate neighbors of each atom in the simulation system.^42−44^ Using this algorithm, we define the first hydration shell as containing water molecules with at least one atom neighboring the protein and the second hydration shell as containing water molecules neighboring those in the first hydration shell. Figure 3a shows the LK_7_β protein (yellow) surrounded by water in the first (red) and second (blue) hydration shells in addition to all the water molecules in the simulation system (black). We can then calculate the chiral SFG spectra of the first and second hydration shells, shown respectively as red and blue spectra in Figure 3b, where the spectrum of all water molecules (black, same as in Figure 2c) is also presented for comparison. Remarkably, the spectrum of the first hydration shell almost overlaps with the spectrum arising from all water molecules in the molecular model. For the spectrum arising from all water molecules, the area under the curve of the first hydration shell spectrum accounts for ∼92% of the analogous area. By contrast, the second hydration shell produces almost no signal. Hence, nearly all the chiral SFG response arises from water molecules in the first hydration shell. These results indicate that chiral SFG spectroscopy is sensitive to the water structures in the first hydration shell of the protein at the air–water interface.

### Chiral SFG Response of Water Reveals Local Interactions in the First Hydration Shell

**Figure 4:**
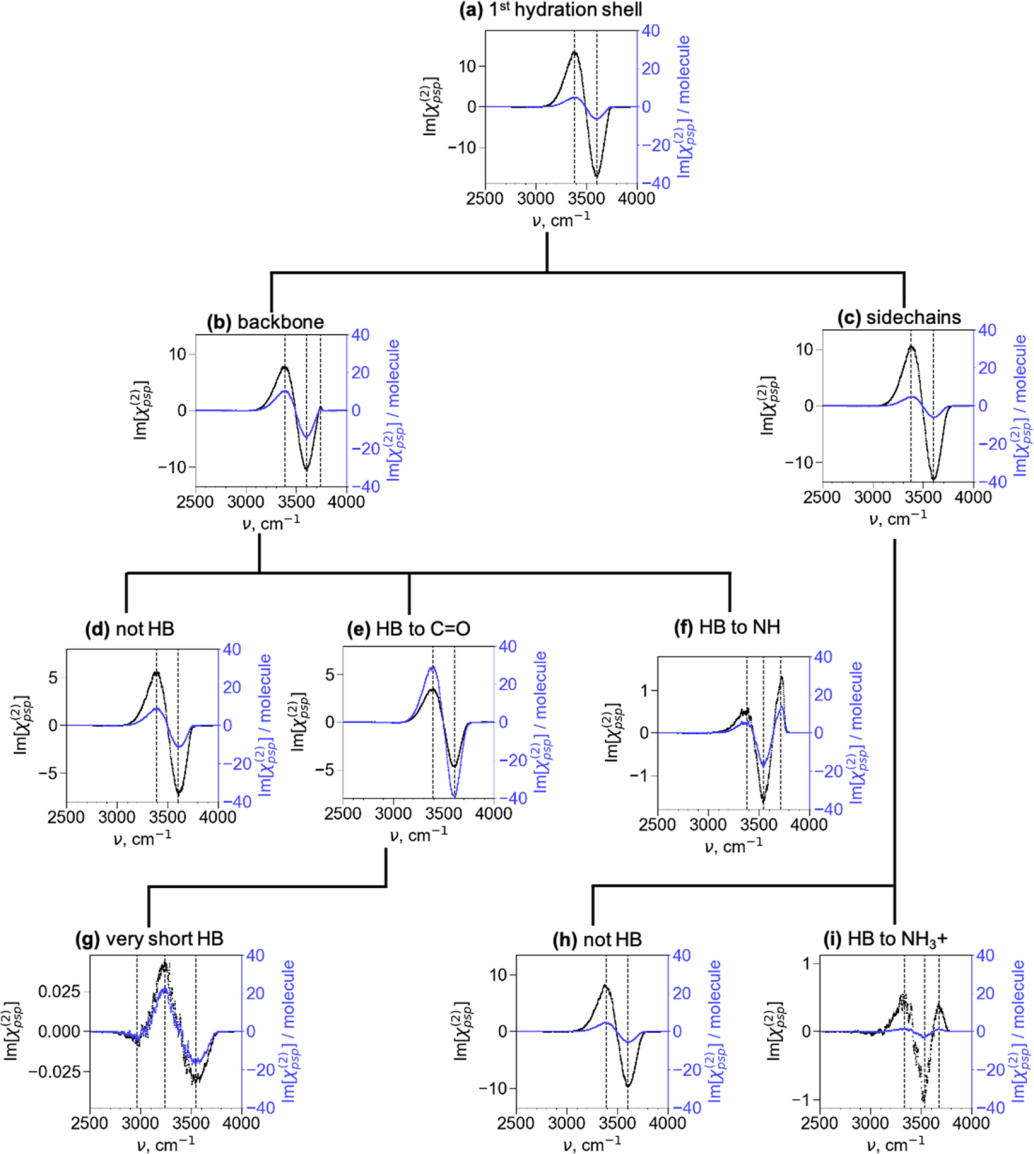
Chiral SFG lineshapes of different subsets of water molecules around (L-) LK_7_β. The names of the plots for panels b and c indicate the region around the protein from which water molecules were taken for each subset. For panels d–i, HB stands for “hydrogen bonded”. “HB to NH” refers to the water accepting a hydrogen bond from an amide N–H group. “HB to C=O” refers to the water donating a hydrogen bond to an amide carbonyl group. The left *y*-axes and the black spectra indicate the absolute signals to show the relative intensities of the various spectra. The right *y*-axes and the blue spectra indicate the chiral SFG signal *per water molecule* for each subset.

We further analyze the chiral SFG signals generated from the first hydration shell. We aim to identify the water molecules that generate the chiral SFG responses corresponding to the three spectral regions (∼3050–3150, ∼3300–3400, and ∼3500–3800 cm^–1^) revealed by the experiments (Figure 2a). We then focus on the correlation between the O–H stretching frequencies and the local H-bonding interactions between water and the protein. We examine this correlation because water–water and water–protein H-bonding interactions modulate the O–H stretching frequency, giving rise to chiral SFG responses across the three spectral regions. Specifically, stronger H-bonds with water acting as the H-bond donor lead to lower O–H stretching frequencies. To perform this analysis, we divided the first hydration shell into various subsets of water molecules and computed the chiral SFG spectra of these subsets (Figure 4). Figure 4a shows the spectrum of all water molecules in the first hydration shell. These water molecules are divided into those close to the protein backbone (Figure 4b) and side chains (Figure 4c) (see the Supporting Information for the selection details). Next, the backbone subset was subdivided into the section *not* H-bonded to the protein (Figure 4d) and the section H-bonded to the C=O (Figure 4e) and N–H (Figure 4f) groups. Similarly, the side chain subset (Figure 4c) is grouped into the section *not* H-bonded to the protein (Figure 4h) and the section H-bonded to the positively charged lysine side chains (Figure 4i). To explore the origin of the low-frequency experimental signals at 3050–3150 cm^–1^ (Figure 2a), we further calculated the spectrum of water molecules that formed strong (short) H-bonds with the backbone C=O groups (Figure 4g). All these spectra are plotted with absolute signals (black spectra) on the left axis and signals normalized to the number of water molecules (blue spectra) on the right axis, as shown in Figure 4.

We first focus on the spectra in Figure 4 that share the same line shape as the spectrum of the first hydration shell (Figure 4a), namely, a positive peak at ∼3400 cm^–1^ and a negative peak at ∼3600 cm^–1^. These spectra include Figure 4b–e and h. The full hydration spectrum (Figure 4a) is the sum of the backbone (Figure 4b) and side chain (Figure 4c) spectra. The absolute contributions of these two subset spectra are on the same order of magnitude. Hence, both the protein backbone and side chains can template water molecules into chiral water superstructures and generate the overall chiral SFG signals. Within the backbone subset, water molecules that are not H-bonded to the backbone (Figure 4d) and are H-bonded to the backbone C=O groups (Figure 4e) dominate the spectral response and contribute almost equally to the overall backbone spectrum (Figure 4b). In contrast, within the side chain subset, water molecules that do not engage in H-bonding interactions (Figure 4h) are responsible for almost all the signal in the overall side chain spectrum (Figure 4c). Hence, there are three major contributors to the overall chiral SFG signal of water O–H stretches: water molecules near but not H-bonded to the backbone (Figure 4d), those H-bonded to C=O on the backbone (Figure 4e), and those near but not H-bonded to the side chains (Figure 4h). These subsets of water are likely responsible for the experimental signals of water O–H stretches in the middle and high frequency regions of ∼3300–3400 and ∼3500–3800 cm^–1^, respectively (Figure 2a).

It may be surprising that subsets of water molecules with different molecular interactions with the protein give similar spectral line shapes. Importantly, water molecules in the subsets that are not H-bonded to the protein still participate in H-bonding networks with neighboring water molecules. Indeed, we find that the average number of H-bonds formed by first hydration shell water molecules is 3.2 for those near but not H-bonded to the backbone, 3.2 for those H-bonded to C=O on the backbone, and 3.1 for those near but not H-bonded to the side chains. These numbers are slightly lower than but comparable to an average of 3.4 H-bonds per water molecule when considering all water molecules in the entire system. For water molecules in these H-bonding environments, the overall line shape appears to be simply the signature of water experiencing electric fields from the protein, and it does not matter whether the H-bond acceptor is a carbonyl group oxygen or a water oxygen because the electric fields experienced by the O–H groups are similar. This typical H-bonding environment for most water molecules around the antiparallel β-sheet likely accounts for the general line shape of water molecules in the first hydration shell of LK_7_β.

Three spectra in Figure 4 deviate from the overall two-peak line shape. Among them, two exhibit similar spectral features: the backbone NH-bound (Figure 4f) and the side chain NH_3_^+^-bound (Figure 4i) spectra. These spectra show not only the general feature with a positive peak at ∼3400 cm^–1^ and a negative peak red-shifted to around ∼3550 cm^–1^ but also an additional positive peak at ∼3700 cm^–1^. A water O–H stretch at that high of a frequency often suggests a weak H-bonding or H-bond-free environment. Indeed, the sharp high-frequency peak in Figure 4f resembles the prototypical dangling O–H pointing toward the air at the air–water interface, which was first reported by Shen and co-workers.^45^ Analysis of our MD trajectories indicates that about 15% of the water molecules that are H-bonded to backbone N–H have OH groups pointing into the vacuum. These OH groups in H-bond-free environments are expected to contribute to the high-frequency signal. In contrast, the positive peak at ∼3700 cm^–1^ in the side chain NH_3_^+^-bound spectrum (Figure 4i) likely arises from a different source. The NH_3_^+^-bound water molecules form only 2.3 H-bonds on average (versus 3.4 for all water molecules in the system), suggesting that the H-bonding structure of water near the positive charge of the lysine is severely disrupted. This disruption was previously proposed to give rise to a high-frequency peak corresponding to a water molecule containing O–H groups not engaged in H-bonds.^45^

The last spectrum that shows a line shape different from the general one is Figure 4g. This spectrum is generated from water molecules that form the shortest (strongest) H-bonds with the backbone C=O (1.6 Å or less between the water hydrogen and protein oxygen). The spectrum contains three peaks. Two of these peaks resemble the general positive peak (3400 cm^–1^) and negative peak (3600 cm^–1^) of the first hydration shell spectrum (Figure 4a) but are red-shifted to 3250 and 3550 cm^–1^, respectively. These red shifts are often associated with stronger H-bonds. Uniquely, the spectrum also shows a small but significant third peak at ∼3000 cm^–1^. A peak at this low of a frequency is also present in the spectrum of all water molecules in the system (inset, Figure 2c). Such low-frequency signals are seen in the H_2_^18^O isotope substitution experiments (Figure 2b). Hence, the low-frequency signals could potentially be due to water molecules strongly H-bonded to carbonyl groups on the protein backbone. Nonetheless, it is important to note that Fermi resonance with the first overtone of the water bending mode can also generate a water vibrational response at ∼3200 cm^–1^.^41,46^ Because our current calculations do not include Fermi resonance, further investigation is needed to better understand the experimentally observed signals in the low-frequency region of ∼3050–3150 cm^–1^.

### Chiral SFG Intensity and Water Mobility are Correlated for Water H-Bonded to a Protein

**Figure 5:**
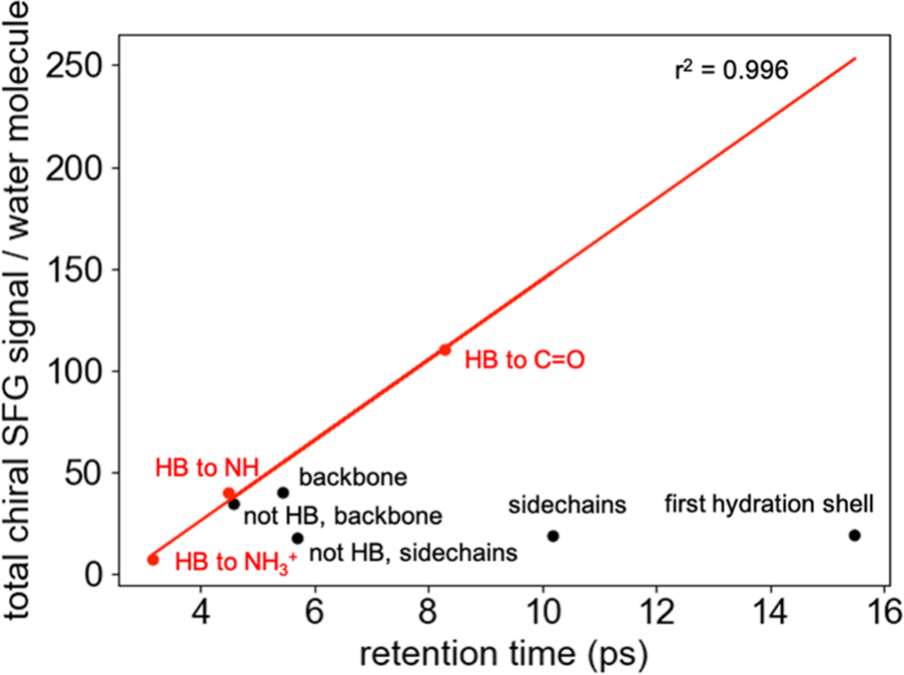
Relationship between water mobility and chiral SFG signal generation. This plot depicts the chiral SFG signal per water molecule versus the average retention time of a water molecule in each subset shown in Figure 4.

We further investigated how water mobility regulates the chiral SFG intensity. We hypothesized that the more static the water molecules in the first hydration shell, the higher the chiral O–H stretching signals produced per water molecule in that subset. To test this hypothesis, we calculate the retention times (*i.e*., the average time spent within a subset) of the water molecules in each subset described in Figure 4.^47^Figure 5 plots the chiral SFG signal per water molecule versus the retention time. The overall correlation is noticeably poor. However, there is a positive correlation between the retention time and the chiral SFG signal production efficiency per water molecule for water molecules that interact with the protein through H-bonds. These water molecules belong to the subsets that are hydrogen bonded to backbone C=O, backbone N–H, and side chain NH_3_^+^ groups (*i.e.*, “HB to C=O”, “HB to N–H”, and “HB to NH_3_^+^” in Figure 4). The above correlation analyses reveal two classes of water molecules in the first hydration shell that generate chiral SFG signals. The first class is characterized by water molecules that form H-bonds with the protein. In this case, the more a water molecule’s mobility is reduced by H-bonding interactions with the protein, the more it produces a chiral signal (Figures 4e, f, and i and 5). The second class is characterized by water molecules that do not form H-bonds with the protein (Figures 4d and h and 5). According to this analysis, the water molecules that form more persistent H-bonds with the protein (*i.e.*, H-bonds that are retained for longer times prior to exchange) contribute the most to the chiral signal.

### Chiral SFG Theory Can Explain the Selectivity of Chiral SFG to the First Hydration Shell

**Figure 6:**
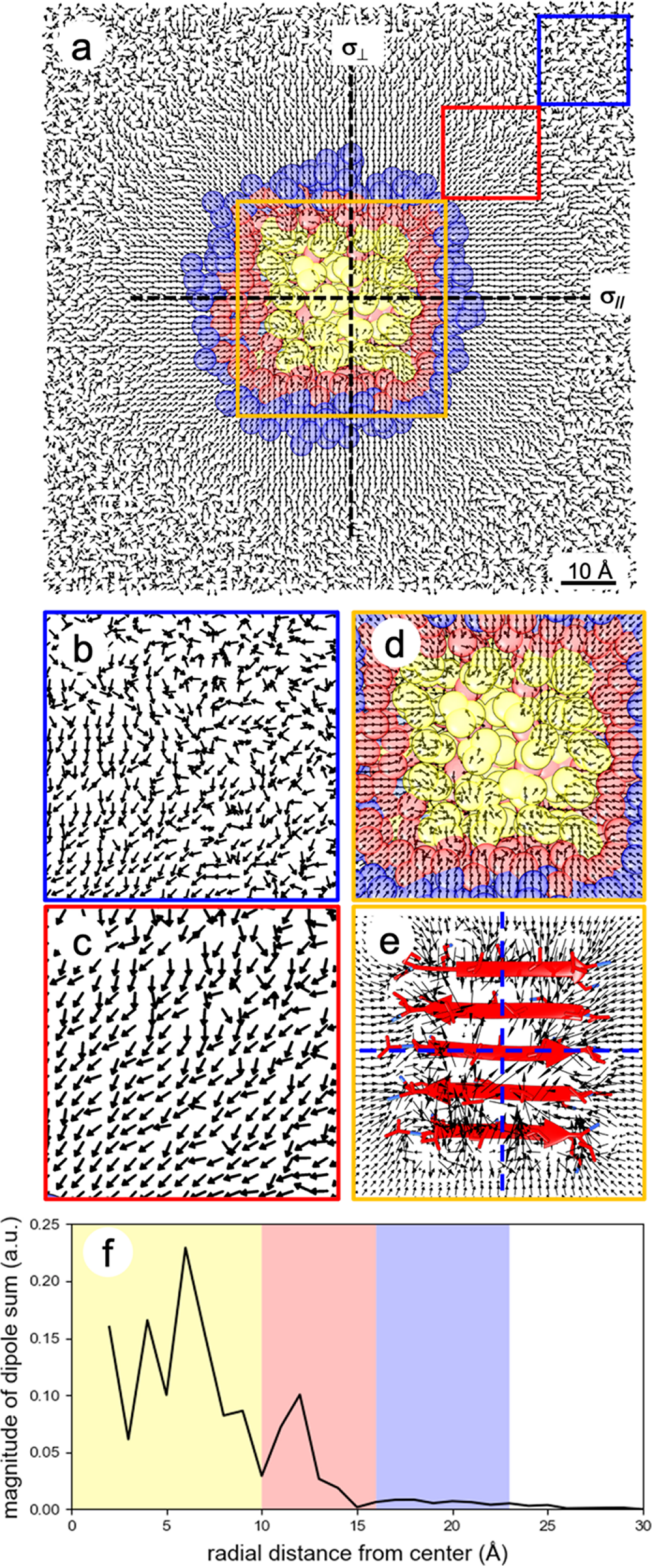
Symmetrical and asymmetrical orientations of water molecular dipole moments outside and inside the first hydration shell of the LK_7_β protein, respectively, at the vacuum–water interface. Water dipoles bisect the H–O–H angle and point toward the water oxygen. (a) Top view of water dipoles at the vacuum–water interface. The protein structure of LK_7_β (yellow) is the average conformation over the course of the MD trajectory. Red and blue regions, respectively, indicate the first and second hydration shells. Water dipoles point toward the protein due to the positive charges of the lysine residues. (b–d) Zoomed-in details of water dipoles in the blue, red, and orange boxes, respectively. (e) Zoomed-in details of water dipoles in the orange box, where the magnitudes of water dipoles are represented by the relative lengths of the arrows. For water dipoles outside the first hydration shell but still within ∼40 Å of the protein, two reflection planes perpendicular to the interface, namely, σ_∥_ aligning with and σ_⊥_ perpendicular to the β-strands, can be identified. For water dipoles inside the first hydration shell, no such reflection planes can be identified. (f) The magnitude of the vector sum of the water dipoles is plotted as a function of radial distance from the center of Figure 6a.

We then sought to elucidate the origin of the selectivity of chiral SFG to the first hydration shell. We questioned whether the ordering of water dipoles by the LK_7_β protein extends beyond the first hydration shell and, if it does, why this extended ordering does not generate chiral SFG signals. Figure 6a shows the top view of the simulation system composed of LK_7_β at the vacuum–water interface, where the protein (yellow) is surrounded by the first hydration shell (red) and the second hydration shell (blue). The average water molecular dipole moments in the interfacial plane were calculated with a resolution of 1 Å and are presented as unit vectors in Figures 6a–d. To emphasize even a slight bias in the dipole direction, the relative magnitude of each vector is not depicted in these figures. Figure S6 provides a depiction with the actual vector magnitudes, illustrating that the ordering of water dipoles past the second hydration shell is minimal compared to the ordering near the protein.

Figure 6a shows that the water dipoles point toward the protein because LK_7_β contains positively charged lysine side chains. Clearly, LK_7_β changes the ordering of water molecules past the first hydration shell. This change in ordering extends 30–40 Å away from the protein (red box, Figure 6c) and gradually fades into random arrangements beyond 40 Å (blue box, Figure 6b). Focusing on the water molecules closest to the protein, Figure 6d (orange box) depicts the orientations of water dipoles inside the first hydration shell, and Figure 6e depicts the average water dipole in this region with both direction and magnitude. The magnitude at a given gridpoint can be less than the dipole moment of one water molecule due to averaging over different orientations and the occupation. The arrangement of water dipoles within the first hydration shell appears to be less symmetrical than the arrangement of water dipoles beyond the first hydration shell, mainly due to direct interactions between the water molecules and the chiral protein.

The ordering pattern shown in Figure 6 can explain the remarkable sensitivity of chiral SFG to the first hydration shell of LK_7_β in terms of the symmetry-based chiral SFG theory.^33^ The theory developed by Simpson in 2004 dictates that a surface-specific chiral SFG response is allowed from an interface of uniaxial C_∞_ symmetry. In this context, the lack of a reflection plane perpendicular to the interface is the hallmark of a chiral interface, making it distinct from an achiral interface with *C*_∞*v*_ symmetry. The absence of a reflection plane perpendicular to the interface is key to distinguishing chiral SFG from conventional (achiral) SFG methods in terms of providing selectivity to interfacial chirality. Figure 6a shows that the ordering beyond the first hydration shell is striking but highly symmetrical. At least two reflection planes of the water dipoles can be identified, namely, σ_∥_ aligned with the β-strands and σ_⊥_ perpendicular to the β-strands (Figure 6a). Hence, water molecules outside the first hydration shell are not in *C*_∞_ symmetry and thus are not chiral SFG-active. In contrast, the ordering of water dipoles in the first hydration shell is much less symmetrical. No reflection plane can be identified in any direction, which is more evident in the plot of the magnitudes of the water dipole moments (Figure 6e).

An alternative illustration of the lack of symmetry in the first hydration shell is presented in Figure 6f. This plot shows the magnitude of the sum of the water dipole moment vectors within each 1 Å annulus between concentric circles in the *xy*-plane, originating at the center point. A nonzero magnitude of this sum is a good indication of asymmetry (chirality). The yellow, red, and blue regions roughly correspond to the area of the protein, the first hydration shell, and the second hydration shell, respectively. The region beyond the first hydration shell shows little or no magnitude, indicating radial symmetry across the two reflection planes (σ_||_ and σ_⊥_); thus, the water arrangement is achiral in the *C*_∞*v*_ symmetry. The region composed of the protein and the first hydration shell shows a significant magnitude of the dipole vector sum; hence, the water arrangement is chiral in the *C*_∞_ symmetry, providing chiral SFG sensitivity.

We performed the same analysis for water dipoles below the water surface up to 14 Å (Figure S5) and made the same observation. Water molecules in the first hydration shell form a supramolecular structure with the uniaxial C_∞_ symmetry and thus are chiral SFG-active. Water molecules beyond the first hydration shell, however, are organized in the *C*_∞*v*_ symmetry and hence are chiral SFG-inactive. Consequently, the selectivity of chiral SFG to the first hydration shell can be elucidated using the chiral SFG theory.^33^

## Conclusions

This study establishes the label-free selectivity of chiral SFG for probing vibrational structures of water in the first hydration shell of proteins. The selectivity of chiral SFG is based on chirality transfer from a protein to the first hydration shell. However, proteins also impact the water orientation and presumably dynamics beyond the first hydration shell (Figure 6). Many label-free physical methods that can detect the hydration of biomolecules report on water well beyond the first hydration shell. For example, intermolecular nuclear Overhauser effect NMR can report on water molecules 4–5 Å from the surface of biomolecules, but the signal can be dominated by couplings between protein and water molecules far outside the first hydration shell.^48^ Terahertz spectroscopy measures collective vibrations of water molecules averaged over water molecules both near and far from the protein surface.^49,50^ Although 2D infrared spectroscopy is more sensitive to localized protein vibrations and can provide site-specific insights into short-range protein–water interactions, the technique lacks selectivity for the O–H stretch of water molecules in protein hydration shells.^51^ X-ray crystallography and electron microscopy provide a high level of site specificity for the interaction of water with biomolecules, but these techniques are sensitive to the most strongly associated water molecules and lack reliable data about more dynamic water populations.^52^ Neutron scattering is selective for the first hydration shell; however, the method requires powder samples or highly concentrated protein solutions,^51^ restricting its use in applications for which *in situ* or interfacial measurements are desired. Conventional (*achiral*) SFG has been used to probe the hydration of proteins and biomolecules at interfaces. Nonetheless, this method detects all water molecules oriented by biomolecules (see Figure 6) and lacks selectivity to the first hydration shell.^53−58^

The higher selectivity of *chiral* SFG for the first hydration shell will be beneficial for answering many outstanding questions in molecular biology concerning both intramolecular and intermolecular interactions of biomolecules. For instance, the structural rearrangements and dynamical fluctuations of biomolecules that enable all molecular functions^5,59,60^ rely on the presence of water in ways that are still not understood. Most importantly, the chemical details underlying the role of the first hydration shell in the hydrophobic effect, protein denaturation, and protein folding remain enigmatic. With chiral SFG, the architectures and dynamics of hydration shells involved in protein–protein, protein–ligand, protein–nucleic acid, and protein–lipid interactions, including transmembrane proteins in amphiphilic lipid environments, can be investigated. To tackle such important biophysical problems, chiral SFG methods rely on the synergy between chiral SFG experiments and computational analysis, as demonstrated in this study. This is especially true for extracting chiral SFG vibrational lineshapes that are hidden in the experimental spectrum (Figure 4). Such analyses will prove useful for relating the chiral SFG responses of water to specific water–protein or protein–protein interactions.

Chirality transfer from small molecules to the solvent has been observed in prior studies,^61^ but the exceptional sensitivity of chiral SFG to the chirality of the first hydration shell around biomolecules at interfaces presents unique opportunities for exploring questions relevant to the origin of life. Hydration, homochirality, and interfaces are defining features of life. Understanding these aspects in the context of the origin of life^62−65^ demands a technique that can detect chirality at interfaces. Water may have played a role in the emergence of homochirality through the creation of extended chiral molecular structures beyond the immediate borders of chiral molecules.^31,63,66^ Furthermore, prebiotic chemistry such as formation of peptide bonds may have taken place in microdroplets^67^ at the interface formed between water and air or between water and an organic phase. Our discovery that chiral SFG exclusively selects the first hydration shell around biomolecules at interfaces allows chiral SFG to tackle new questions and produce new insights about the emergence of biological life.

## Methods

### Sample Preparation

(L-) LK_7_β (GL Biochem Ltd., Shanghai, China) and (D-) LK_7_β (AnaSpec, Inc., Fremont, CA) were obtained as lyophilized powders and dissolved in H_2_O (or H_2_^18^O) at a concentration of 1 mM. The peptide solutions (10 μL) were applied to the right-handed *z*-cut α-quartz surface. The solution was dried in a desiccator under a nitrogen flow to prevent the exchange of ambient humidity with H_2_^18^O in the hydrated protein film. Spectra were collected immediately.

### Phase-Resolved Vibrational Chiral SFG

The phase-resolved chiral SFG spectra reported in this study were recorded using a broadband SFG spectrophotometer, previously described.^68^ Chiral SFG recordings utilized the *psp* polarization (*p*-polarized sum frequency, *s*-polarized visible, *p*-polarized infrared). Spectra were collected along the +*y* and −*y* axes of the quartz calibrated to the laboratory frame (see the Supporting Information).^31^ For all reported spectra, 10–12 spectra (2 min each) were acquired along both the +*y* and −*y* axes and averaged. The average spectra along the +*y* and −*y* directions were normalized by the signal from the clean quartz surface and subtracted as follows:^69,70^
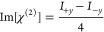
1

Obtaining the phase-resolved vibrational chiral SFG spectrum of LK_7_β from 2900–3800 cm^–1^ with our femtosecond broadband SFG spectrophotometer required data collection in two spectral windows (Figure S1). The frequencies of the SFG spectra in both spectral windows were calibrated using a polystyrene standard (Buck Scientific; 0.05 mm film). The individual spectra were overlaid, normalized to the N–H stretching peak of LK_7_β (∼3270 cm^–1^), and stitched to produce the overall spectrum (see the Supporting Information).

### MD Simulations

Five LK_7_β (LKLKLKL) strands were arranged into an antiparallel β-sheet that was solvated in TIP4P-Ew water^71^ (a version of the TIP4P model adapted for the Ewald summation), with at least 1 nm of solvent on each side of the protein. The upper half of the water was then removed, and chloride ions were added to neutralize the system, leaving a slab with two vacuum–water interfaces, one of which contained the protein. The *z*-dimension of the system was extended to create a vacuum region equal in size to the slab. Periodic boundary conditions were applied in three dimensions. Acetyl groups were added to the N-termini of the peptides, and amide (−NH_2_) groups were created at the C-termini to stabilize the β-sheet. After energy minimization, the slab was equilibrated in the *NVT* ensemble for 6 ns at 298 K using Langevin dynamics (friction coefficient of 1 ps^–1^ and time step of 1 fs). The ff14SB force field was used for the protein.^72^ The particle-mesh Ewald method^73^ was used to treat long-range electrostatic interactions. The SETTLE algorithm^74^ was used to maintain rigid water molecules, and the SHAKE method^75^ was used to constrain bonds involving hydrogens. For the production run, Langevin dynamics was propagated for 100 ns, and a configuration was stored every 10 fs. OpenMM 7.4 tools^76^ were used for equilibration and production runs as well as for part of the system setup, and VMD scripts were used to arrange the antiparallel β-sheet. An NVIDIA Tesla V100 GPU and CUDA 9.2 were used to run the MD simulations. The Supporting Information contains details regarding the equilibration. The trajectory was not wrapped to put the protein in the center for the SFG calculations, as the calculation takes periodic boundary conditions into account.

### Calculation of SFG Spectra of Subsets of Water Molecules

The inhomogeneous limit version of Skinner’s electric field mapping approach^36,38−40^ was used to calculate the spectra, as in our previous work.^31,32^ SFG signals are derived from the second-order susceptibility (χ^(2)^) of a system. Chiral SFG is distinct from the more commonly reported achiral SFG because the two techniques probe different elements of χ^(2)^ by manipulating the polarization of the incoming radiation and the detector in different ways. The form of chiral SFG presented here (*e.g.*, *psp*, see above) detects the orthogonal tensor component χ_*zyx*_^(2)^, which is only nonzero for chiral systems at the interface, whereas achiral SFG (*e.g.*, *ssp*) detects nonorthogonal components such as χ_*yyz*_^(2)^, which can be nonzero at any interface.^21^ In the calculations, we controlled the output element of χ^(2)^ by analyzing particular components of the dipole and polarizability.^77^ This is analogous to changing the polarization of the incoming radiation and detector in experiments. Previous work by Simpson^33^ and Konstantinovsky et al.^32^ provided a quantitative description of the ability of certain *achiral* molecules (such as water) to produce *chiral* SFG signals when in a chiral superstructure.

In the construction of the exciton Hamiltonian, intermolecular couplings between O–H groups on different molecules were neglected to allow the clear separation of water molecule subsets, but intramolecular couplings between O–H groups on the same molecule were included.^32^ All water subset selections were done using an in-house code. The MDAnalysis tool select_atoms was used extensively for simple selections.^78^ See Figure S4 for a graphical illustration of our approach.

### Using Voronoi Tessellation to Identify Water Molecule Neighbors

The first hydration shell was defined using Voronoi tessellation. Voronoi tessellation of a set of points consists of cells containing the regions closer to each point than to any other point in the set. We broke down the atoms of the system into Voronoi cells. Water neighbors of the protein are defined as water molecules for which at least one atom’s Voronoi cell is in contact with a Voronoi cell belonging to a protein atom.^79^ The freud library^79^ was used to access the Voronoi tessellation engine voro++^80^ from Python to efficiently produce Voronoi tessellations and neighbor lists. Periodic boundary conditions were taken into account by all water selections, including Voronoi tessellation. Within the Voronoi diagram, the water molecules bordering a protein cell were considered the “first hydration shell”, while the water molecules bordering the first hydration shell were considered the “second hydration shell”. The “backbone” and “side chain” subsets were deliberately made to overlap somewhat (∼46 water molecules shared between them in a typical selection) to ensure that all water molecules in the first Voronoi shell were considered. As a result, the number of water molecules in the two subsets adds up to slightly more than the number of water molecules in the first hydration shell. The hydrogen-bonding criteria used in this work are a distance between heavy atoms less than 3.5 Å and a hydrogen-bonding angle greater than 135°. The distance cutoff was chosen to be larger than that typically used to ensure that water molecules *not* hydrogen bonded to the protein were highly unlikely to be engaging in any form of specific interaction with the protein.

### Calculation of Local Water Dipoles on a Grid

The average water dipole moments were obtained by adding the dipoles of the water molecules at each grid point (based on the location of the oxygen) over the trajectory to determine the total dipole at each grid point and subsequently normalizing this vector to obtain the unit dipole vector. The grid point calculation was done as described previously.^31^ A 100 ns trajectory was used to generate the images in Figure 4, with frames considered every 1 ps for a total of 100 000 frames analyzed.

### Analysis of Retention Time

A 100 ns trajectory was used to generate these data, with frames saved every 1 ps for a total of 100 000 frames analyzed. The plotted retention time, with a time resolution of 1 ps, was the average time from the arrival of a water molecule into a given subset (selection) to its exit from that subset.

## Supporting Information Available

Description of the SFG spectrometer, fitting of experimental spectra, quartz-only spectrum (experimental), details of MD equilibration, details of selection methods for water subsets, information about the construction of the exciton Hamiltonian for the calculation of SFG spectra, illustration of the lack of a reflection plane in the first hydration shell at various distances from the interface, and unnormalized (nonunit) dipole moment vectors around LK_7_β (PDF)
