# Supplementary material for: Detecting the First Hydration Shell Structure around Biomolecules at Interfaces
Source: ACS Cent Sci. 2022 Sep 6;8(10):1404–14. doi: 10.1021/acscentsci.2c00702 (PMC9615115; doi:10.1021/acscentsci.2c00702)
Supplement: Supplementary file 1 — oc2c00702_si_001.pdf [file oc2c00702_si_001.pdf]

Supporting Information

Detecting first hydration shell structure around biomolecules at interfaces

Daniel Konstantinovsky[1,2†], Ethan A. Perets[1†], Ty Santiago[3,‡], Luis Velarde[3], Sharon Hammes-Schiffer[1]*, and Elsa C. Y. Yan[1]*

[1]Department of Chemistry, Yale University, New Haven, CT 06520, USA
[2]Department of Molecular Biophysics and Biochemistry, Yale University, New Haven, CT 06520, USA
[3]Department of Chemistry, University at Buffalo, Buffalo, NY 14260, USA

[‡]Current address: Department of Chemistry, Yale University, New Haven, CT 06520, USA
† These authors contributed equally

*Corresponding Authors
Email Addresses: sharon.hammes-schiffer@yale.edu, elsa.yan@yale.edu

Contents

Description of the SFG spectrometer......S2
Experimental spectra: Sewn spectra......S3
Experimental spectra: Fits......S4
Quartz-only spectrum......S7
MD equilibration details......S7
Selection methods for water subsets......S8
Construction of the Hamiltonian for calculation of the SFG spectrum......S10
Illustration of the lack of a reflection plane in the first hydration shell......S11
References......S12

Description of the SFG spectrometer

A detailed description and characterization of the custom SFG spectrometer utilized in the studies can be found in Ma, *et al.*[1] Briefly, a 5 kHz ultrafast regenerative amplifier (Spitfire, Spectra-Physics) is pumped by two Nd:YLF lasers (Empower, Spectra-Physics) and seeded with a Ti:Sapphire laser (MaiTai, Spectra-Physics). The amplifier creates 120 femtosecond pulses with a total power of 6 W. A 50/50 beamsplitter is used to divide the amplifier output along two paths. The first path (3 W) is passed through a custom pulse-shaper yielding narrow-band pulses centered at 800 nm. The second path (3 W) is used to pump a tunable optical parametric amplifier (TOPAS, Spectra-Physics/Light Conversion) to generate broad-band infrared pulses. The resultant visible and infrared beams are spatially and temporally overlapped at the sample surface. The SFG signal reflected from the sample interface is dispersed with a monochromator, and the spectrum is collected with a CCD. The polarization of the SFG, visible, and infrared beams for all experiments reported in the main text was *p*-polarized, *s*-polarized, and *p*-polarized, respectively.[2]

Experimental spectra: Sewn spectra

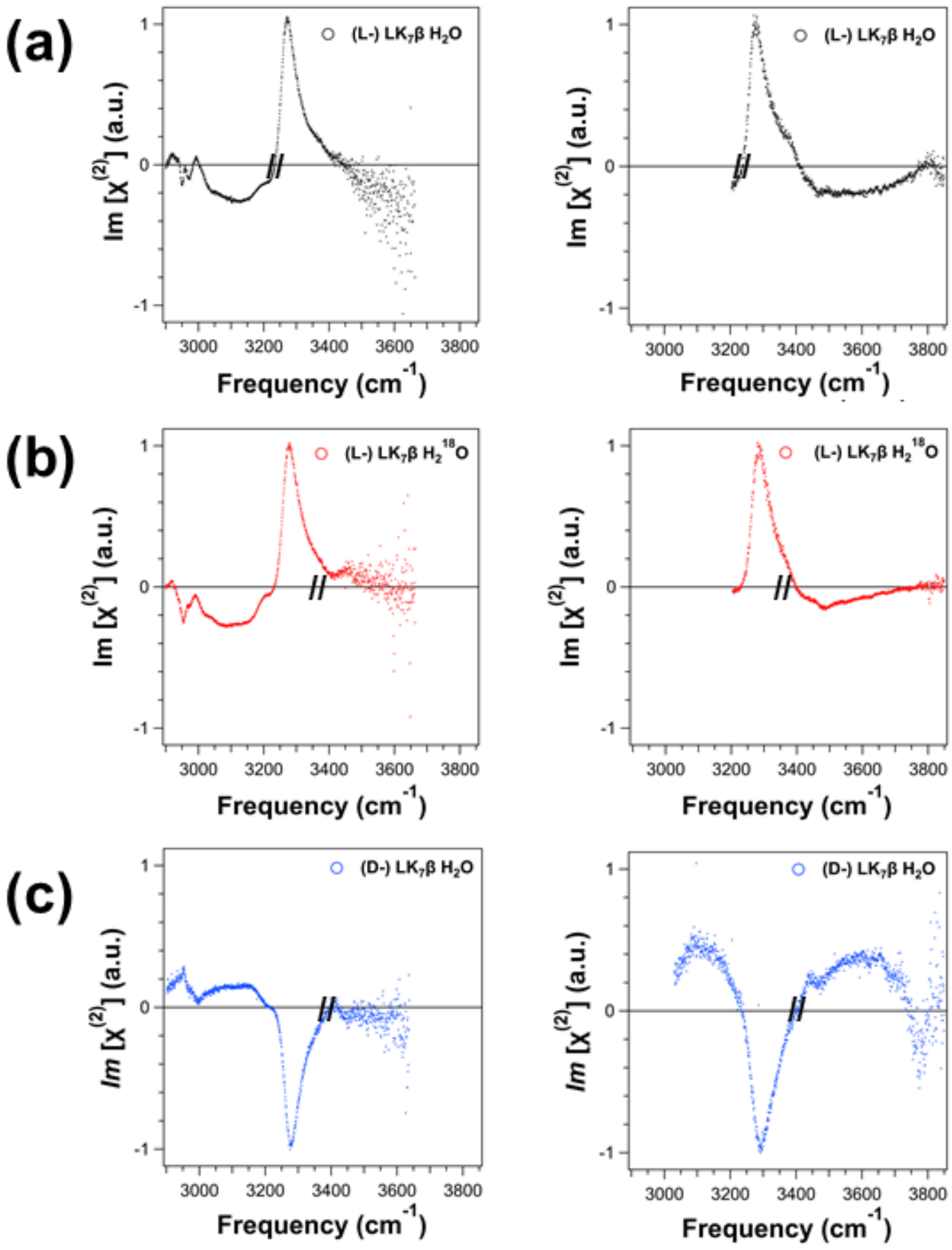

Figure S1. The two spectral windows collected for each spectrum presented in Figure 2 in the main text. The two individual spectra were overlaid and normalized to the N-H stretching peak of LK$_7$β at ~3270 cm^{-1} and stitched together at the point indicated with hashmarks. The noisy regions that appear at the high frequency ends of the spectra are due to normalization of the broadband spectra by clean quartz. a.u.: arbitrary units.

Experimental spectra: Fits

Description of Fitting Experimental Spectra.

In the spectral region 2900 – 3800 cm^{-1}, C-H and N-H stretching of the LK$_7$β protein, O-H stretching of water, and possibly additional signals due to Fermi resonance of water can contribute to the vibrational spectra (for more information on vibrational analyses of proteins, see Barth and Zscherp).[3] The experimental spectra in Figure 2b were fit with Lorentzians according to:

$$\mathrm{Im}[\chi^{(2)}] \propto \mathrm{Im}[\sum_q \frac{A_q}{\omega_{IR} - \omega_q - i\Gamma_q}] \tag{S1}$$

where ω_q is the resonant frequency of the qth vibrational mode, A_q is the amplitude, Γ_q is the half-width half-maximum of the qth vibrational mode, and ω_{IR} is the frequency of the incident infrared. Therefore, each vibrational peak contributes three parameters (ω_q, A_q, Γ_q). The minimum number of vibrational peaks needed to fit the experimental data (Figure 2b) is determined by residual analyses as shown in Figure S2. Eleven peaks are used to fit the spectra, and they are tentatively assigned as shown in Table S1.

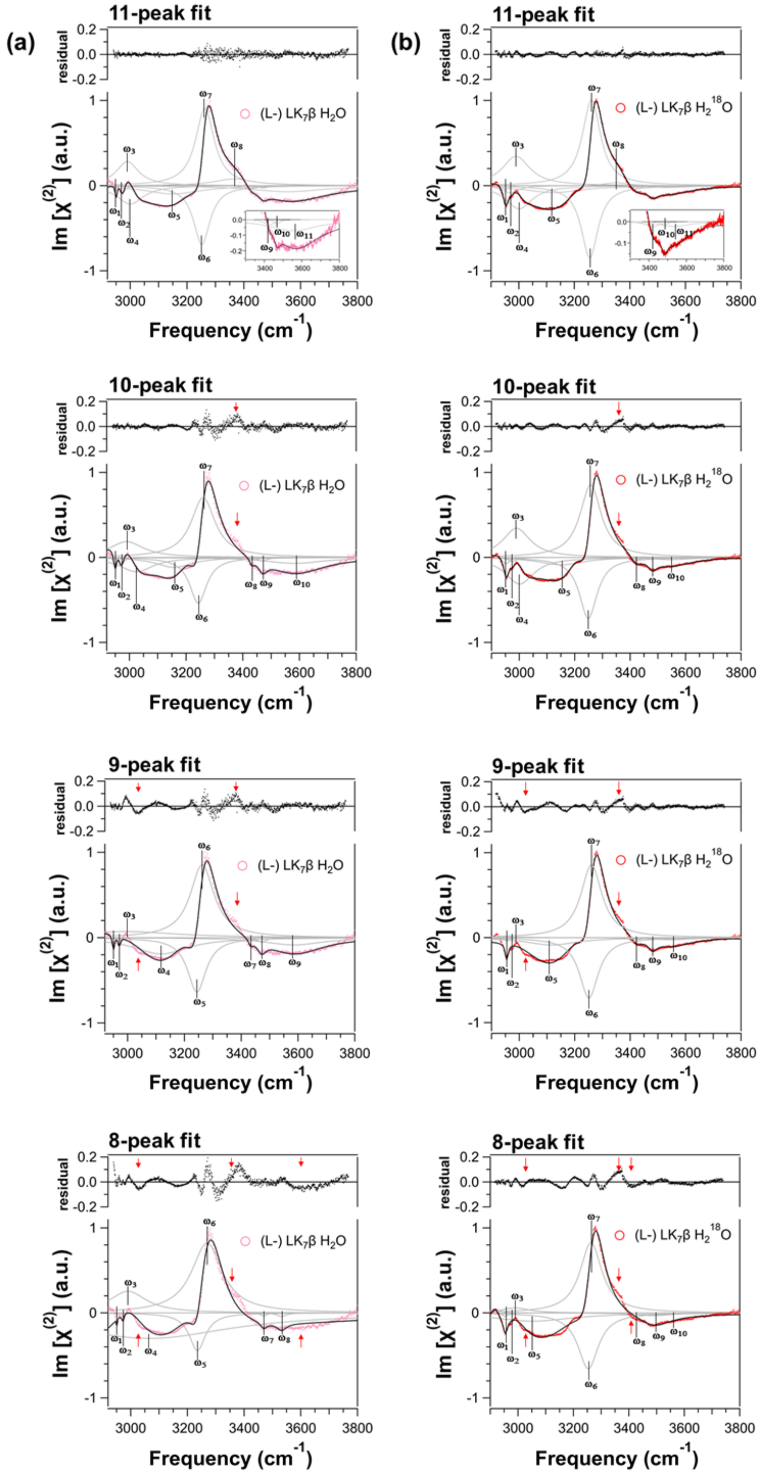

Figure S2. Fitting and residual analyses of the fitting to the experimental spectra of (a) $LK_7\beta$-H_2O and (b) $LK_7\beta$-$H_2{}^{18}O$ presented in Figure 2b in the main text with (from top to bottom) 11, 10, 9, and 8 vibrational peaks. Fitting parameters for the 11-peak fitting (top row) are reported in Table S1. Individual vibrational peaks of each fitting are shown as gray lines (these have been scaled to fit within the viewing window). Residuals between the experimental data and the fitting are included above each spectrum. The residual analyses suggest that 11 component vibrational resonances are needed to model the experimental spectra in Figure 2b of the main text.

Parameter	Figure 2b (L-) LK$_7$β H_2O	Figure 2b (L-) LK$_7$β $H_2{}^{18}O$	Tentative Assignments
ω_1	2950 ± 1	2952 ± 1	C-H (LK$_7$β)
A_1	-0.7 ± 0.2	-2.8 ± 0.9	
Γ_1	4.2 ± 0.7	12.5 ± 1.6	
ω_2	2973 ± 1	2970 ± 2	C-H (LK$_7$β)
A_2	-3.1 ± 0.6	-13.1 ± 12.2	
Γ_2	13.1 ± 1.3	34.3 ± 9.1	
ω_3	2990 ± 0*	2990 ± 0*	C-H (LK$_7$β)
A_3	40.7 ± 0*	94.1 ± 35.6	
Γ_3	48.0 ± 2.6	60.7 ± 0.0*	
ω_4	3000 ± 2	3001 ± 7	C-H or N-H (LK$_7$β)
A_4	-36.7 ± 3.2	-71.1 ± 19.7	
Γ_4	56.6 ± 4.4	58.0 ± 1.5	
ω_5	3151 ± 5	3125 ± 4	O-H (H_2O) and/or F.R.** with H_2O bend overtone
A_5	-35.0 ± 7.4	-46.3 ± 10.3	
Γ_5	119.5 ± 16.8	125.7 ± 17.6	
ω_6	3252 ± 1	3255 ± 1	N-H (LK$_7$β)
A_6	-74.1 ± 2.9	-139.6 ± 2.3	
Γ_6	35.9 ± 0.6	36.6 ± 0.4	
ω_7	3263 ± 1	3261 ± 1	N-H (LK$_7$β)
A_7	108.8 ± 0.0*	179.1 ± 0.0*	
Γ_7	40.0 ± 0.7	39.6 ± 0.5	
ω_8	3366 ± 6	3350 ± 2	O-H (H_2O)
A_8	20.3 ± 4.0	7.8 ± 2.1	
Γ_8	85.9 ± 10.0	50.1 ± 6.6	
ω_9	3426 ± 2	3420 ± 10	N-H (LK$_7$β)
A_9	-3.2 ± 1.6	-4.5 ± 0.8	
Γ_9	31.2 ± 7.6	40.9 ± 7.8	
ω_{10}	3468 ± 2	3483 ± 1	N-H (LK$_7$β)
A_{10}	-1.6 ± 0.2	-1.4 ± 0.2	
Γ_{10}	20.0 ± 0.0*	20.0 ± 0.0*	
ω_{11}	3555 ± 10	3538 ± 6	O-H (H_2O)
A_{11}	-34.4 ± 1.8	-9.5 ± 1.5	
Γ_{11}	161.1 ± 12.2	95.6 ± 7.5	

*Parameters held during fitting.
**F.R. = Fermi Resonance

Table S1. The fitting parameters (see equation S1, above) used for the 11-peak fitting shown in Figure S2. Water contributions are suggested based on $H_2{}^{18}O$ substitution.

Quartz-only spectrum

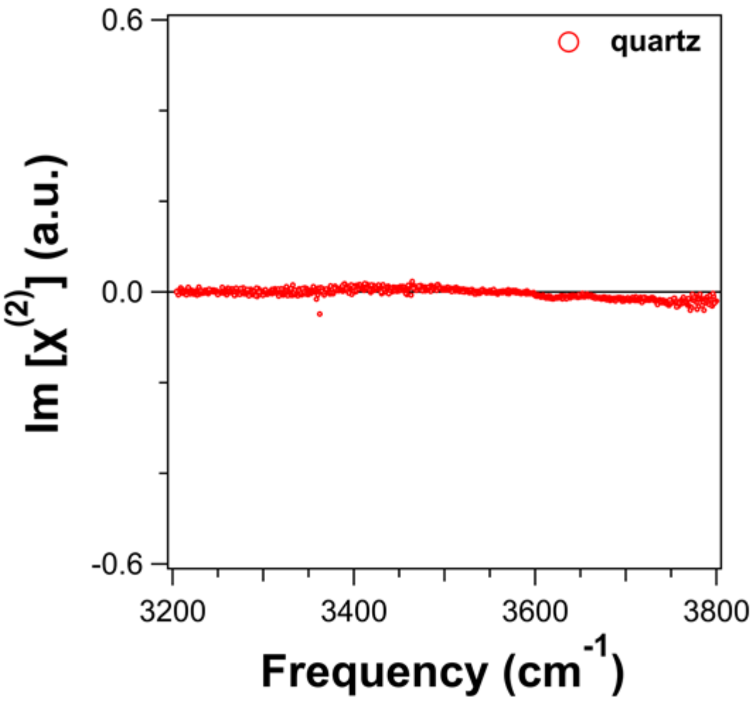

Figure S3. Chiral SFG spectrum of clean quartz substrate in the absence of $LK_7\beta$. The spectrum is normalized by GaAs. The spectrum shows no vibrational resonances. 2 mins. acquisition time. a.u.: arbitrary units.

MD equilibration details

See the Methods section in the main text for more information about the MD simulations. The equilibration was initiated with an energy minimization of the solvent. This was followed by a 500 ps NVT equilibration of the solvent with a restrained protein (force constant of 500 kcal mol^{-1} $Å^{-2}$). Then the positions of the hydrogen atoms of the protein were minimized. Subsequently, the system was minimized three times, gradually reducing the restraints on the protein heavy atoms (100, then 50, then 10 kcal mol^{-1} $Å^{-2}$), followed by energy minimization of the entire system. This minimization was followed by simulated annealing over 360 ps from 0 K to 298 K under NVT conditions, followed by 6 ns of NVT equilibration at 298 K. No NPT equilibration was done because of the vacuum-water interface.

Selection methods for water subsets

The subsets in Figure 4 were selected using a combination of MDAnalysis selection logic,[4] in-house code, and the Voronoi tessellation library freud,[5] which uses the voro++ engine[6] to efficiently generate Voronoi diagrams. All selections were restricted to water molecules in the first hydration shell. All hydrogen bonds were identified if the donor-acceptor heavy atom distance was less than 3.5 Å and the donor-hydrogen-acceptor angle was greater than 135°. This cutoff distance is larger than typically used for defining hydrogen bonds because we wanted to ensure that the non-hydrogen-bonded subsets were truly free from hydrogen-bonding interactions with the protein. Periodic boundary conditions were taken into account for all water selections, including the Voronoi tessellations and hydrogen bonding analyses.

Figure 4a (entire first hydration shell)

The freud library was used to generate a Voronoi diagram with points corresponding to all atoms in the system. The neighbor list feature was then used to identify water molecules with at least one atom's Voronoi cell bordering the Voronoi cell of at least one protein atom. All atoms in the water molecules were considered as possible neighbors to the protein.

Figure 4b (backbone)

Backbone-associated water molecules were selected using the following MDAnalysis selection logic: *byres (resname HOH and (around 4.5 ((not resname HOH) and (name N H CA HA C O HN1 HN2))) and (not around 3.0 ((resname LEU) and not name N H CA HA C O HN1 HN2) or ((resname LYS) and not name N H CA HA C O HN1 HN2)))*

This selection was fairly liberal in terms of backbone-associated water molecules but ensured that no water molecules closely associating with the sidechains were selected.

Figure 4c (sidechains)

Sidechain-associated water molecules were selected using the following MDAnalysis selection logic: *byres (resname HOH and (around 4.5 ((resname LYS) and not name N H CA HA C O HN1 HN2) or ((resname LEU) and not name N H CA HA C O HN1 HN2)) and (not around 3.0 (not resname HOH) and (name N H CA HA C O HN1 HN2)))*

This selection was fairly liberal in terms of sidechain-associated water molecules but ensured that no water molecules closely associating with the backbone were selected.

Figure 4d (backbone – not hydrogen bonded to protein)

First, molecules were preselected using the backbone MDAnalysis selection logic. Then, all water molecules hydrogen bonded to the C=O, NH, or NH_3^+ groups on the protein were identified and excluded, and the remaining water molecules not hydrogen bonded to the protein were considered in this selection. Both acceptor and donor scenarios were considered for hydrogen bonds with NH, although water donor configurations that met our hydrogen bonding definition were rare. The terminal NH_2 groups were also considered as NH groups.

Figure 4e: (backbone – hydrogen bonded to C=O)

First, molecules were preselected with the following MDAnalysis selection logic to reduce the number of water molecules needing to be processed by our hydrogen bond identification code (to improve performance with no effect on the result): *byres ((resname HOH) and (around 3.5 (not resname HOH NA CL Na Cl)))*. Then, water molecules donating a hydrogen bond to a C=O group were identified.

Figure 4f: (backbone – hydrogen bonded to NH)

The same procedure was followed as above with C=O groups, except that water molecules *accepting* hydrogen bonds from NH groups were considered. The water molecule donor scenario was not considered after it was found that these configurations are rare and produce a negligible SFG signal.

Figure 4g: (backbone, hydrogen bonded to C=O with very short hydrogen bonds)

First, water molecules hydrogen bonded to C=O were identified. Then, the following MDAnalysis selection was used to identify water molecules forming very short hydrogen bonds to the C=O groups: *byres ((resname HOH) and (around 1.6 (not resname HOH NA CL Na Cl)))*.No water molecules were found to form such short hydrogen bonds to any other functional group on the protein.

Figure 4h (sidechains – not hydrogen bonded to protein)

The same procedure was followed as for the backbone-associated not hydrogen bond water molecules, except the sidechain selection logic was used.

Figure 4i (sidechains – hydrogen bonded to lysine NH_3^+)

The same procedure was followed as for the C=O groups and NH groups, except water molecules accepting hydrogen bonds from NH_3^+ groups were identified.

Construction of the Hamiltonian for calculation of the SFG spectrum

The exciton Hamiltonian for the SFG spectrum calculation was constructed as illustrated in Figure S4. The main idea is to treat only the selected water molecules in the exciton Hamiltonian and to regard those water molecules, the other water molecules, and the protein as point charges influencing the local electric field, which in turn leads to transition dipoles and polarizabilities for each selected O-H group. All water selections began by selection of the first hydration shell (see Methods). The number of water molecules in the selection could fluctuate slightly between frames without negatively impacting the calculation because the calculation used the inhomogeneous limit approximation, where the signal is conceived as a simple average of individual frames.

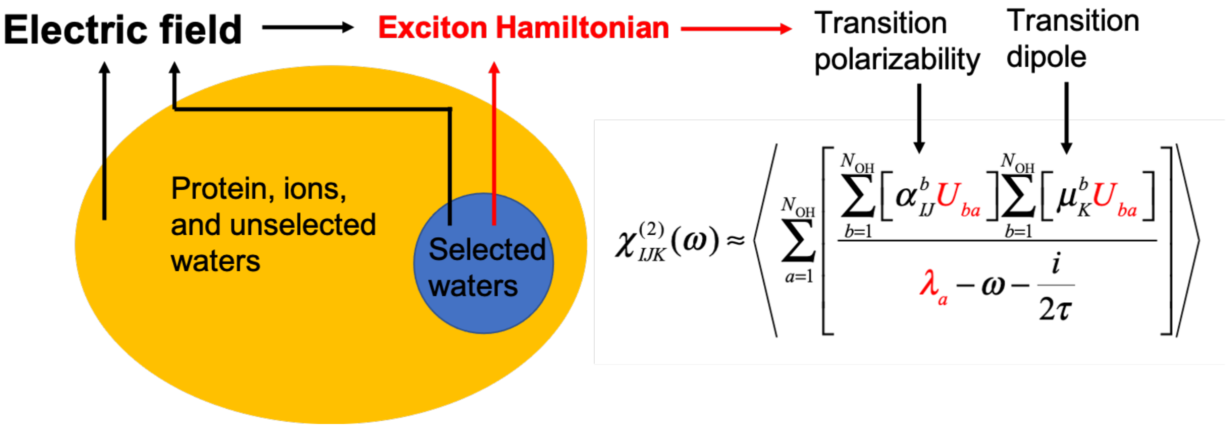

$$\chi_{IJK}^{(2)}(\omega) \approx \left\langle \sum_{a=1}^{N_{OH}} \left[\frac{\sum_{b=1}^{N_{OH}} \left[\alpha_{IJ}^{b} U_{ba} \right] \sum_{b=1}^{N_{OH}} \left[\mu_{K}^{b} U_{ba} \right]}{\lambda_a - \omega - \frac{i}{2\tau}} \right] \right\rangle$$

Figure S4. A schematic of our adaptation of Skinner's electric field mapping approach to calculate SFG spectra arising from subsets of the water molecules in the system. Here, $\boldsymbol{\alpha}$ is the transition polarizability tensor, $\boldsymbol{\mu}$ is the transition dipole vector, $\mathbf{U}$ are the eigenvectors of the Hamiltonian, and $\boldsymbol{\lambda}$ are the eigenvalues. τ is the vibrational lifetime (1.3 ps here).

Illustration of the lack of a reflection plane in the first hydration shell

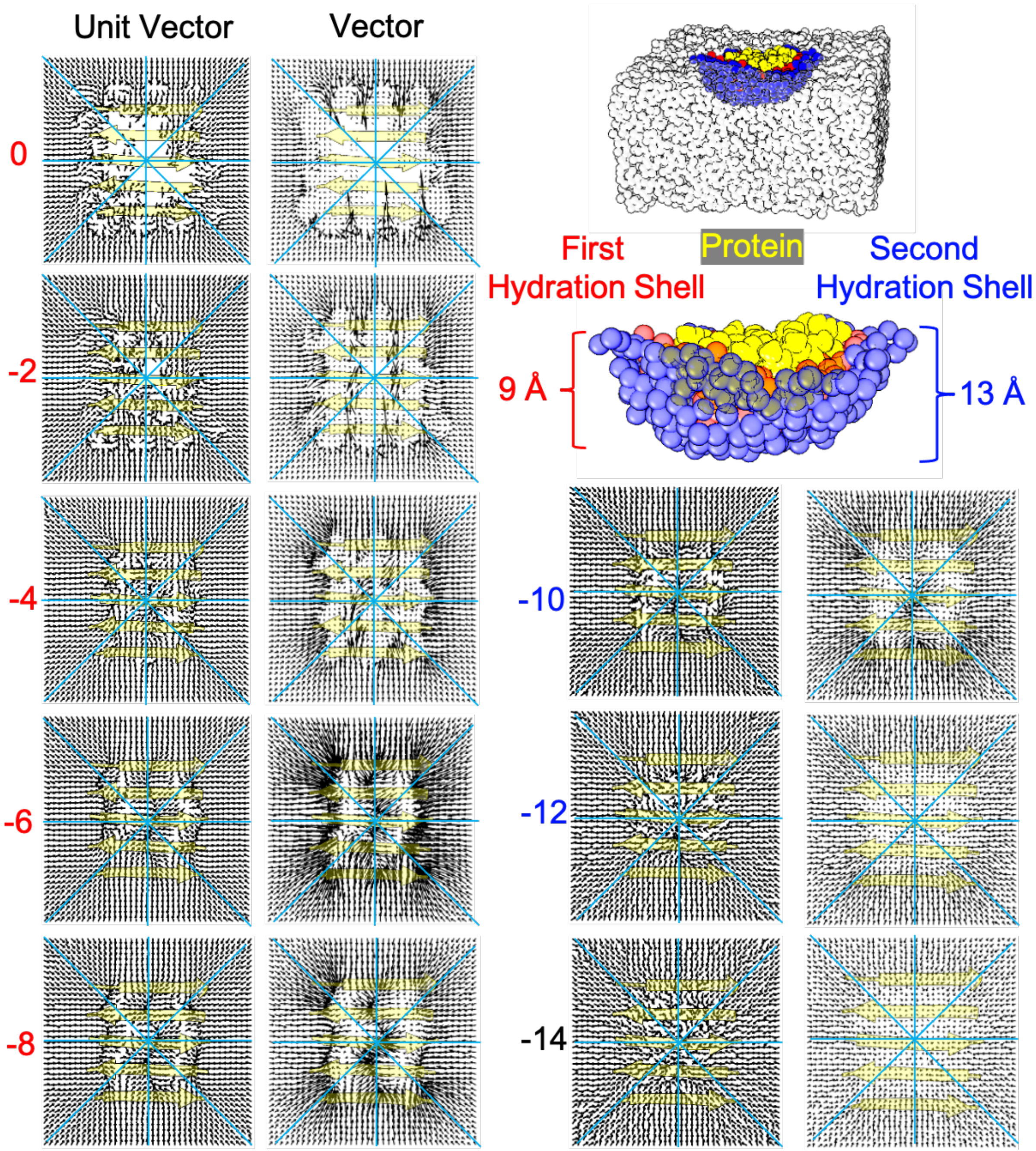

Figure S5. Water dipole moment vector directions at various depths (in Å) from the vacuum-water interface. The LK$_7$β protein is shown in yellow. The blue lines are a guide for the eye. This figure shows that the lack of reflection plane perpendicular to the vacuum-water interface seen in Figure 6 in the main text extends to the bottom of the first hydration shell (~ –8 Å). The water dipole pattern becomes completely symmetric only at –12 to –14 Å, but the greatest asymmetry is between 0 and –8 Å. Both unit vector and vector with magnitude representations are shown.

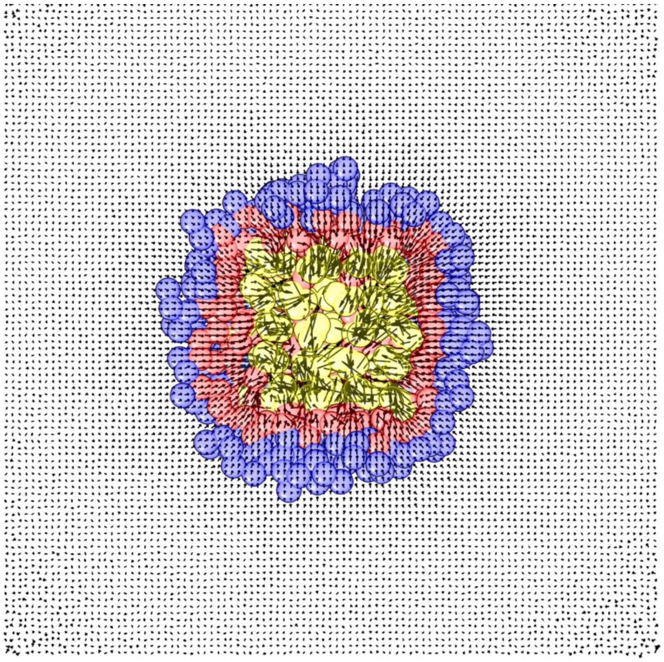

Figure S6. Unnormalized relative magnitudes of the dipole moment vectors at various locations around LK$_7$β. The ordering of water dipoles past the second hydration shell is minimal relative to the ordering near the protein (see Figure 6 in the main text for comparison).

References

1. Ma, G.; Liu, J.; Fu, L.; Yan, E. C. Y. Probing Water and Biomolecules at the Air—Water Interface with a Broad Bandwidth Vibrational Sum Frequency Generation Spectrometer from 3800 to 900 cm−1. *Appl. Spectrosc.* **2009,** *63*, 528-537.
2. Yan, E. C. Y.; Fu, L.; Wang, Z.; Liu, W. Biological Macromolecules at Interfaces Probed by Chiral Vibrational Sum Frequency Generation Spectroscopy. *Chem. Rev.* **2014,** *114*, 8471-8498.
3. Barth, A.; Zscherp, C. What vibrations tell about proteins. *Quarterly Reviews of Biophysics* **2002,** *35*, 369-430.
4. Michaud-Agrawal, N.; Denning, E. J.; Woolf, T. B.; Beckstein, O. MDAnalysis: A toolkit for the analysis of molecular dynamics simulations. *J. Comput. Chem.* **2011,** *32*, 2319-2327.
5. Ramasubramani, V.; Dice, B. D.; Harper, E. S.; Spellings, M. P.; Anderson, J. A.; Glotzer, S. C. freud: A software suite for high throughput analysis of particle simulation data. *Comput. Phys. Commun.* **2020,** *254*, 107275.
6. Rycroft, C. H. VORO++: A three-dimensional Voronoi cell library in C++. *Chaos* **2009,** *19*, 041111.
